# Enhancing Surface Modification and Carrier Extraction in Inverted Perovskite Solar Cells via Self-Assembled Monolayers

**DOI:** 10.3390/nano14020214

**Published:** 2024-01-19

**Authors:** Gisung Kim, Hyojung Kim, Mijoung Kim, Jaegwan Sin, Moonhoe Kim, Jaeho Kim, Haoran Zhou, Sung Ho Kang, Hye Min Oh, JungYup Yang

**Affiliations:** 1 Korea Institute of Fusion Energy (KFE), Daejeon 34133, Republic of Korea; kgs@kunsan.ac.kr; 2 Department of Physics, Kunsan National University, Gunsan 54150, Republic of Korea; kmj0602@kunsan.ac.kr (M.K.); sjk329595@kunsan.ac.kr (J.S.); moonhoekim@kunsan.ac.kr (M.K.); dolrans@kunsan.ac.kr (J.K.); 3 The Institute of Basic Science, Kunsan National University, Gunsan 54150, Republic of Korea; hyojungkim@kunsan.ac.kr; 4 Renewable Energy Materials Laboratory (REML), Advanced Institute of Convergence Technology (AICT), Seoul National University, Suwon 16229, Republic of Korea; zhouharoan@snu.ac.kr

**Keywords:** perovskite solar cells, self-assembled monolayers, hole-transporting layer, surface modification, thermal stability

## Abstract

Perovskite solar cells (PSCs) have been significantly improved by utilizing an inorganic hole-transporting layer (HTL), such as nickel oxide. Despite the promising properties, there are still limitations due to defects. Recently, research on self-assembled monolayers (SAMs) is being actively conducted, which shows promise in reducing defects and enhancing device performance. In this study, we successfully engineered a p-i-n perovskite solar cell structure utilizing HC-A1 and HC-A4 molecules. These SAM molecules were found to enhance the grain morphology and uniformity of the perovskite film, which are critical factors in determining optical properties and device performance. Notably, HC-A4 demonstrated superior performance due to its distinct hydrophilic properties with a contact angle of 50.3°, attributable to its unique functional groups. Overall, the HC-A4-applied film exhibited efficient carrier extraction properties, attaining a carrier lifetime of 117.33 ns. Furthermore, HC-A4 contributed to superior device performance, achieving the highest device efficiency of 20% and demonstrating outstanding thermal stability over 300 h.

## 1. Introduction

Perovskite solar cells (PSCs) have experienced a significant increase in efficiency, from 3.8% in 2009 to 26.1% in the present day [1,2]. These solar cells are typically composed of a perovskite light-absorbing layer sandwiched between an n-type electron transport layer (ETL) and a p-type hole transport layer (HTL) [3,4,5,6]. Upon the incidence of light on the solar cell, an exciton is generated within the active layer, which subsequently separates into electrons and holes. These free carriers are then transported through the ETL and HTL, respectively, to the external electrode [7]. As for HTLs, molecular layers based on organic or conductive polymers have been widely studied to enhance the efficiency of PSCs [8]. However, these types of HTL layers have notable limitations in terms of reliability due to their environmental sensitivity [9,10,11]. As a result, there is an urgent need to explore alternative HTL materials that are not only efficient and stable, but also offer potential scalability and a reduction in overall manufacturing costs. Inorganic compounds with energy levels suitable for PSCs have been investigated. Nickel oxide (NiO), an inorganic HTL, exhibits semiconductor properties, including excellent stability, high hole mobility, a wide band gap (~3.5 eV), high optical transparency, and a solution process coating method, demonstrating its potential for use in PSCs [12,13,14]. However, a reduction in efficiency due to surface recombination, traps, and defects remains a challenge [15]. To address these issues, research on passivation [16,17], additives [18,19,20], and buffer layers [21,22] is being undertaken. Recently, several efforts have been made to reduce defects and enhance the performance of devices using self-assembled monolayers (SAMs) [23]. A SAM molecule is made up of three parts: a head group, a linkage tail and a functional group [24]. The head group attaches directly to the substrate and helps stabilize the surface, using various substances such as silane, carbonyl acid, phosphoric acid, and thiol [25]. The linkage tail connects the head group and functional group, and it affects optical properties and performance, depending on its molecular size and tilt angle [26]. The functional group, which is located on the surface that is exposed, can alter the properties of the surface based on the material used. 

Herein, we fabricated inverted p-i-n PSCs using HC-A1 and HC-A4 molecules. These multifunctional molecules, previously employed in dye-sensitized solar cells, exhibited excellent stability and offered the potential to control charge recombination [27,28]. The impact of introducing an additional layer of HC-A1 and HC-A4 between the perovskite and the transporting layer was investigated, both structurally and optically, using various techniques. The application of HC-A1 and HC-A4 resulted in an improvement in the grain morphology and uniformity of the perovskite active layer. In particular, HC-A4 led to significant surface modification and efficient carrier extraction with its pronounced hydrophilic properties. As a result, HC-A4-applied solar cells exhibited the highest power conversion efficiency (PCE) of 20% with outstanding long-term stability. 

## 2. Materials and Methods 

### 2.1. Materials 

Indium-doped tin oxide (ITO) substrates (15 Ω/sq) were purchased from Nippon Sheet Glass (Osaka, Japan). N,N-dimethylformamide (DMF; 99.8%), dimethyl sulfoxide (DMSO; ≥99.9%), lead(II) bromide (PbBr_2_; 99.9%), and 2-propanol (IPA;99.5%) were purchased from Sigma-Aldrich (St. Louis, MO, USA). Lead(II) iodide (PbI_2_; >98.0%) and cesium iodide (CsI; >99.0%) were purchased from TCI (Tokyo, Japan). Formamidinium iodide (FAI), methylammonium bromide (MABr) were purchased from GreatCell Solar (New South Wales, Australia). Bathocuproine (BCP; 99.5+%) and fullerene (C_60_) were purchased from Nano-C (Westwood, MA, USA), and nanoparticles (NiCT-7, 2.5 wt% NiO in Ethanol) were purchased from Nano Clean Tech (Seoul, Republic of Korea). SAM materials (HC-A1 and HC-A4) were synthesized following previous work [27,28], and ethanol (>99.9%) and tetrahydrofuran (THF; 99%) were purchased from EMSURE® (Merck, Darmstadt, Germany) and JUNSEI (Kyoto, Japan), respectively.

### 2.2. Device Fabrication 

ITO/glass substrates were sequentially cleaned with deionized (DI) water, Hellmanex, acetone, ethyl alcohol, and isopropanol for 20 min and then dried using N_2_ gas. Any remaining organic residuals were then eliminated through a UV ozone surface treatment for 30 min. The NiO nanoparticles were stirred for 30 min, and 50 μL of the filtered NiO nanoparticle solution was spin-coated onto the ITO substrate at 4000 rpm for 40 s, and then annealed at 350 °C for 30 min. The CsFAMAPb(IBr)_3_ (CFM) precursor solution was then prepared by mixing 568 mg of PbI_2_, 79.8 mg of PbBr_2_, 187 mg of FAI, 12.2 mg of MABr, 66 mg of CsI, and 220 mg of DMSO with 755 mg of DMF solution and stirring for 1 h. The precursor solution was then spin-coated in two steps: 500 rpm for 5 s and 5000 rpm for 45 s, with 300 μL of chlorobenzene dropped as an anti-solvent after 15 s into the second step. The resulting perovskite film was annealed at 100 °C for 45 min. All spin-coating processes were conducted within a glove box. Subsequently, a C_60_ layer, electron transporting layer (ETL), was deposited by thermal evaporation under high vacuum (10^−7^ Torr) conditions, achieving a thickness of 15 nm at a deposition rate of 0.5 Å/s. A BCP solution containing 6 mg of BCP in IPA was then spin-coated at 4000 rpm for 30 s and dried at room temperature in an N_2_ atmosphere. Finally, 100 nm Ag electrodes were deposited by thermal evaporation under high vacuum (10^−7^ Torr) conditions, with thicknesses of 10 nm and 90 nm sequentially deposited at rates of 0.3 Å/s and 1.0 Å/s, respectively.

### 2.3. Device Characterization 

The perovskite structure was analyzed using a high-resolution X-ray diffractometer (HR-XRD; Empyrean, London, UK) The absorption and transmittance spectra were obtained using a spectrophotometer (UV-Vis; Hitachi (Tokyo, Japan), UH4150). The top-view and cross-sectional scanning electron microscopy (SEM) images were measured with a field-emission scanning electron microscope (FE-SEM; Hitachi, SU8220). Photoluminescence (PL) spectroscopy was performed using a confocal microscope (NANOBASE (Seoul, Republic of Korea, XperRAM S series) equipped with a 532 nm laser (100 nW). Time-resolved PL spectra were obtained using a 405 nm pulsed laser with a repetition rate of 32 MHz with a 10 s integration time, and an excitation power of 21.3 nW was maintained to prevent light-induced damage to the perovskite sample. Atomic force microscope (AFM) images were measured using an AFM system (NX-10; Park systems, Suwon, Republic of Korea) with a non-contact mode cantilever (PPP-NCHR; NANOSENSORS, Neuchatel, Switzerland). The scan rate was controlled as 0.5 Hz. The current–voltage (*J*-*V*) characteristics were measured under an AM 1.5 G solar illumination (100 mW/cm^2^) using a 450 W Xenon arc lamp and a class AAA solar simulator (Newport, Narragansett Bay, RI, USA) equipped with Keithley 4200S-SCS (Cleveland, OH, USA). Additionally, external quantum efficiency (EQE) measurements were conducted with a 150 W Xenon arc lamp and IQE200 (Newport, USA) across various wavelengths.

## 3. Results and Discussion

### 3.1. Fabrication and Characterization of SAM 

**Figure 1:**
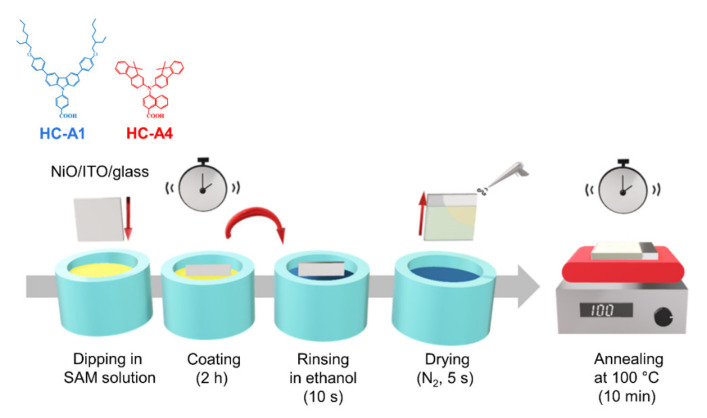
Schematic illustration of the HC-A1 and HC-A4 structures and a detailed SAM bathing process.

Figure 1 illustrates the molecular structure of HC-A1 and HC-A4, along with a detailed fabrication process. The Y-shaped structure consists of a carboxylic acid acceptor linked to benzene (HC-A1) and naphthalene (HC-A4), respectively. The various functional groups of SAM molecules can facilitate charge extraction characteristics between the perovskite and the transporting layer. Solutions of 1 mmol HC-A1 (6 mL ethanol, 4 mL THF) and 1 mmol HC-A4 (10 mL ethanol) were stirred for 1 h, and the NiO nanoparticle-coated substrate was immersed in the SAM solution for 2 h. The SAM-modified substrate was then rinsed in ethanol for 10 s to remove any residual unbounded molecules. Following drying with N_2_ gas, the final substrate was annealed at 100 °C for 10 min.

**Figure 2:**
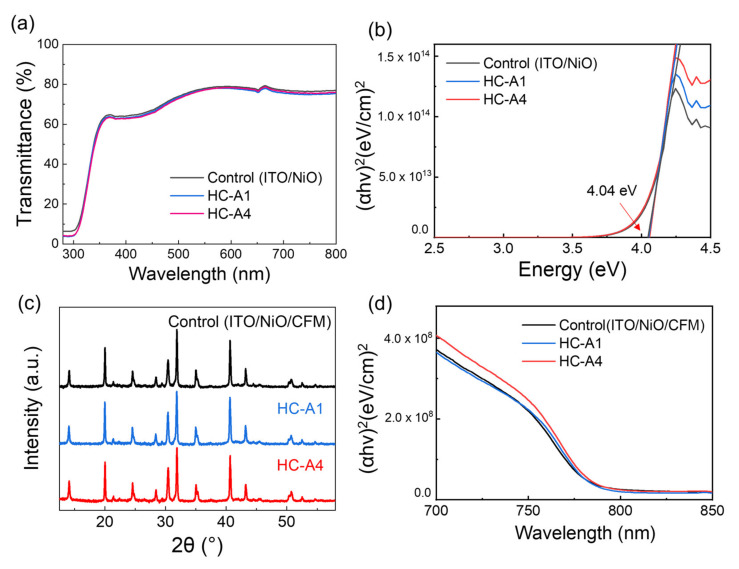
(**a**,**b**) Transmittance and absorption spectra of NiO and SAM-applied NiO on the ITO substrate. (**c**,**d**) XRD and absorption spectra of ITO/NiO/CFM structures, modified with the inclusion of either the HC-A1 or HC-A4 layer.

Figure 2a,b show the transmittance and absorption spectra of ITO/NiO/SAM according to the application of either HC-A1 or HC-A4. Since the additional SAM layer existed on the ITO/NiO substrate, a high transmittance of 60~80% was obtained in the visible range. The optical band gap was determined to be 4.04 eV, both with and without the SAM layer. Subsequently, we explored the characteristics of the perovskite layer with SAM modifications. Figure 2c,d present the XRD and absorption spectra of ITO/NiO/CFM, modified with the inclusion of either HC-A1 or HC-A4 between the NiO and CFM perovskites. In the absence of a SAM layer, the perovskite primarily exhibited diffraction peaks at 14.1° and 20.0°, corresponding to the (001) and (011) planes of CFM. The introduction of the SAM layers resulted in a slight shift of the entire diffraction peaks towards lower angles. Although HC-A1 and HC-A4 molecules are not integrated into the perovskite structure, the diffraction changes can be understood as strain changes, suggesting a slight expansion of the perovskite unit cell due to the addition of the SAM layer below [29]. Consistent with this observation, the optical band gap of CFM perovskite was confirmed at 790 nm (1.57 eV), without noticeable changes following SAM modification. 

### 3.2. Surface Modification via SAM Molecules 

**Figure 3:**
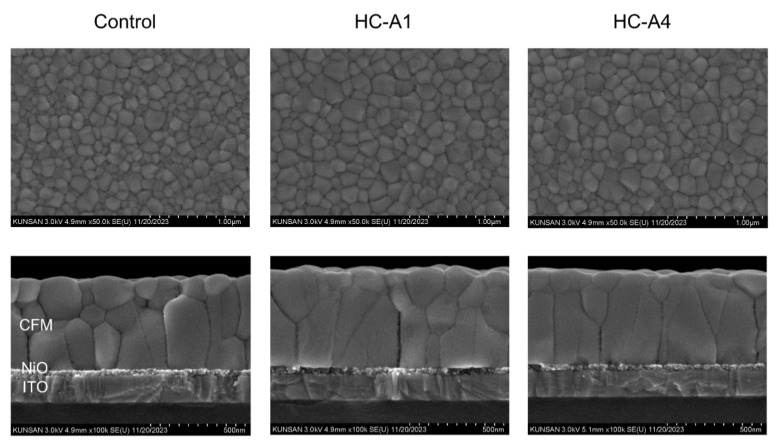
Top-view and cross-sectional SEM images of ITO/NiO/CFM structures, with either HC-A1 or HC-A4 applied between NiO and CFM.

The CFM core structures remained unaffected by the presence of HC-A1 and HC-A4 molecules; however, their grain morphology improved with the addition of the SAM layers. Figure 3 presents the top-view and cross-sectional SEM images of ITO/NiO/CFM structures, both with and without the SAM layers. The scales (1.00 μm and 500 nm) in the SEM images represent the entire width, and the thickness of CFM perovskite was 500 nm across all films. Interestingly, both HC-A1 and HC-A4-applied perovskite films exhibited significant improvement in the vertical CFM grain structure. Additionally, when comparing the control and the HC-A4 samples, an improvement in the grain size on the surface was also observed. We hypothesize that SAM layers, consisting of different functional groups, may alter the surface properties of NiO.

**Figure 4:**
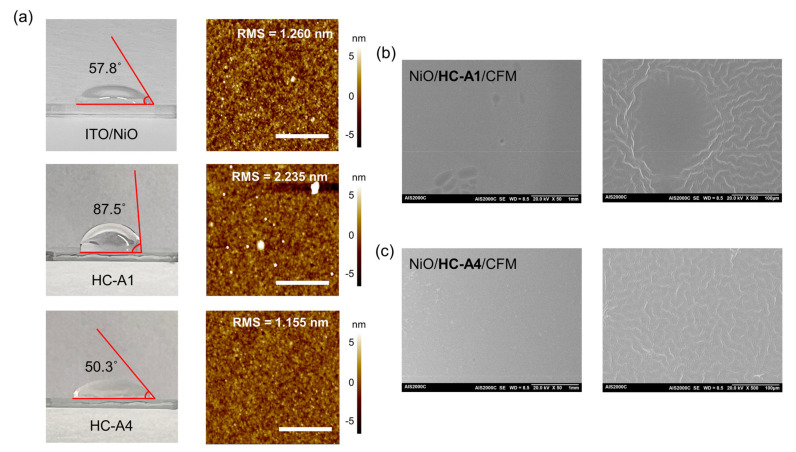
(**a**) Contact angle and AFM (scale bar: 2 μm) images of ITO/NiO/SAM structure, both without and with either HC-A1 or HC-A4. (**b**,**c**) Top-view SEM images of ITO/NiO/CFM structure with SAM molecules between NiO and CFM (scale bar: 1 mm and 100 μm).

Figure 4a,b show the surface difference between HC-A1 and HC-A4 molecules, as evidenced by the contact angle and AFM images. The SAM molecules were applied on the top of the ITO/NiO structure. For the ITO/NiO structure without the SAM layer, the contact angle and root-mean-square (RMS) value were measured to be 57.8° and 1.260 nm, respectively. For HC-A1 and HC-A4, the contact angles were confirmed to be 87.5° and 50.3°, respectively, placing both molecules within the hydrophilic region. However, HC-A4 demonstrated more pronounced hydrophilic properties, attributable to its functional groups, compared to the contact angle of ITO/NiO. The RMS values for the ITO/NiO/SAM structure were also measured, revealing a more uniform surface for HC-A4 compared to HC-A1. The RMS values were determined to be 2.235 nm for HC-A1 and 1.155 nm for HC-A4. Furthermore, we observed a hollow-like CFM surface on the HC-A1 layer through top-view SEM images with low magnification. This observation implies that the perovskite film does not form completely due to the near hydrophobic-like properties of HC-A1. The uniformity of the perovskite film is a critical factor in determining optical properties and device performance; thus, we anticipated that HC-A4, with its hydrophilic properties and smoother surface, could outperform others in all aspects. 

### 3.3. Carrier Extraction via SAM Molecules 

**Figure 5:**
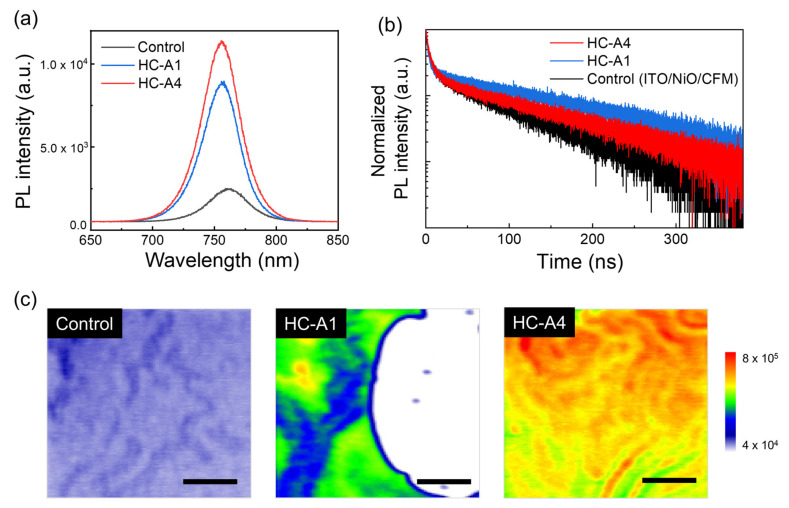
(**a**,**b**) PL and TPRL spectra of ITO/NiO/CFM structures according to the additional SAM molecules. (**c**) PL mapping images of control, HC-A1, and HC-A4 samples, respectively (scale bar: 4 μm).

We measured PL and TRPL spectra of the ITO/NiO/SAM/CFM structures, as shown in Figure 5a,b. The PL intensity was remarkably increased with SAM molecules, attributable to the improved surface and vertical grain morphology. Consistent with this observation, the PL peak position shifted from 761.66 nm to 755.76 nm following SAM modification, suggesting enhanced crystallization and passivated defect level. The TRPL spectra, presented in Figure 5b, were fitted using a tri-exponential function, and the average lifetimes (τ_avg_) were calculated and are listed in Table 1. In comparison to the control sample (73.81 ns), both the HC-A1 and HC-A4 samples demonstrated improved lifetimes of 201.11 ns and 117.33 ns, respectively. This improvement is associated with the quality of perovskite layer in conjunction with the SAM layer. However, it is worth noting that the HC-A4 sample exhibited a shorter lifetime than the HC-A1 sample. A comparison of the TRPL properties with the HTL-including structure revealed the hole-extraction ability of each sample. In other words, HC-A4 transported holes from the perovskite to the NiO layer more efficiently than HC-A1. Moreover, a missing part of PL was observed for the HC-A1 sample in Figure 5c, which is attributed to the hollow-like CFM surface depicted in Figure 4c. Otherwise, the HC-A4-modified film displayed more uniform PL properties than the other samples at the same scale. 

### 3.4. Device Characterization with SAM Molecules 

**Figure 6:**
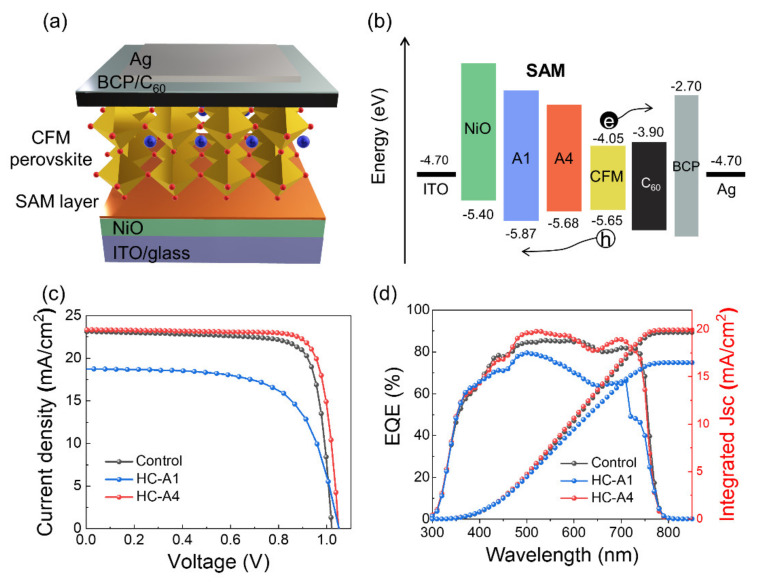
(**a**,**b**) Schematic representation of the inverted solar cell structure and energy level diagram for each layer. (**c**,**d**) *J*-*V* and EQE spectra with integrated *J*_SC_ of control, HC-A1, and HC-A4 devices, respectively.

**Figure 7:**
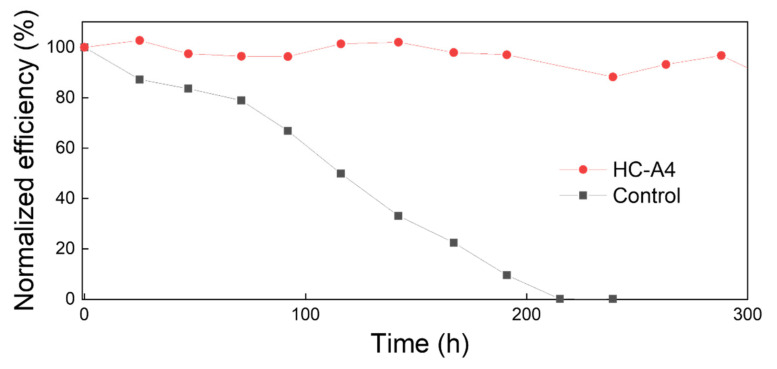
Long-term thermal stability test for the control device and the HC-A4 device at 85 °C in a nitrogen atmosphere.

We fabricated a full p-i-n device structure of ITO/NiO/SAM/CFM/C_60_/BCP/Ag, as depicted in Figure 6a. The energy level diagram for each layer, as illustrated in Figure 6b, shows that the functional group of SAM molecules can facilitate its energy level. The *J*-*V* and EQE curves were measured in Figure 6c,d, and the device parameters are summarized in Table 2. The control device without SAM molecules exhibited a *V*_OC_ of 1.02 V, *J*_SC_ of 23.13 mA/cm^2^, FF of 79.81%, and PCE of 18.81%. Due to its hollow surface, HC-A1 showed the lowest efficiency of 13.04% with poor device *J*_SC_ and FF values. Conversely, HC-A4 device demonstrated enhanced device parameters of 1.05 V, 23.34 mA/cm^2^, 81.80%, and 20.00%, along with the highest integrated *J*_SC_ of 19.88 mA/cm^2^. The uniform surface modification and the rapid charge extraction by the additional HC-A4 layer contribute positively to the device performance. Furthermore, the favorable band alignment of HC-A4 between CFM and NiO, as shown in Figure 6b, can promote hole extraction, thereby enhancing the overall efficiency of the device. Finally, we evaluated the long-term stability of the SAM-modified device and compared it to a control device without SAM, as shown in Figure 7. The stability of both the control and HC-A4 devices was monitored at 85 °C in a nitrogen atmosphere. The performance of the control device degraded rapidly over time and was completely broken down after 200 h. In contrast, the HC-A4 device maintained its initial efficiency for 300 h without significant changes, as a result of the sturdy perovskite crystal structure reinforced by the SAM layer. 

## 4. Conclusions 

In this study, we successfully fabricated a p-i-n perovskite device structure utilizing HC-A1 and HC-A4 molecules. The application of these SAM molecules improved the grain morphology and uniformity of the perovskite film, which are critical factors in determining optical properties and device performance. Notably, HC-A4 outperformed HC-A1 due to its pronounced hydrophilic properties with a contact angle of 50.3°, attributable to its distinct functional groups. Overall, the HC-A4-applied film showed efficient carrier extraction properties, achieving a carrier lifetime of 117.33 ns, and recorded the highest PCE of 20%. Furthermore, the HC-A4 device demonstrated exceptional thermal stability, maintaining its initial efficiency over 300 h. Our study presents HC-A4 as a promising candidate for enhancing device performance, thereby expanding the scope of research on the utilization of SAM molecules in PSCs. 

## Figures and Tables

**Table 1:** TPRL fitting components of ITO/NiO/CFM structures according to the additional SAM molecules.

Sample	A_1_ (%)	τ_1_ (ns)	A_2_ (%)	τ_2_ (ns)	A_3_ (%)	τ_3_ (ns)	τ_avg_ (ns)
Control	0.61	2.537	0.15	14.402	0.19	88.072	73.81
HC-A1	0.77	2.236	0.10	13.647	0.23	213.303	201.11
HC-A4	0.61	2.938	0.21	11.438	0.17	137.043	117.33

**Table 2:** Device parameters of the inverted solar cell structure depending on HC-A1 and HC-A4 applications.

	*V_OC_* (V)	*J*_SC_ (mA/cm^2^)	FF (%)	PCE (%)
Control	1.02	23.13	79.81	18.81
HC-A1	1.05	18.79	66.06	13.04
HC-A4	1.05	23.34	81.80	20.00

## Data Availability

The data that support the findings of this study are available from the corresponding author upon reasonable request.
